# Spontaneous Catheter Fracture Leading to a Retained Fragment After Central Venous Access Port Removal: Should Preoperative Chest X-rays Be Obtained?

**DOI:** 10.7759/cureus.102424

**Published:** 2026-01-27

**Authors:** Alexis Clare, Fuad Turfah

**Affiliations:** 1 General Surgery, Corewell Health, Dearborn, USA

**Keywords:** catheter fracture, central venous access, cxr, mediport, pinch-off syndrome, retained catheter

## Abstract

Central venous access ports are used to administer chemotherapy, antibiotics, and total parenteral nutrition, and for frequent blood draws. The device consists of a subcutaneous reservoir (port) and an intravascular piece (catheter). Access is achieved via direct venipuncture of the subclavian, internal jugular, or cephalic vein with the tip at the cavoatrial junction.

Retained central venous access ports are relatively uncommon. In most cases, an indwelling time greater than two years can cause an adhesive fibrous sheath to form around the catheter, contributing to more difficult removal. Spontaneous fracture of ports is even less common. Our goal is to discuss spontaneous catheter fracture of a central venous access port leading to a retained fragment in an adult. In this paper, we suggest obtaining a preoperative chest X-ray (CXR) in certain patients prior to central venous access port removal and explain the reasoning behind doing so.

## Introduction

A portacath is a type of central venous access device that provides continuous access to a large vein in the body, usually the superior vena cava. It consists of a subcutaneous reservoir attached to a soft, flexible catheter that terminates at the junction of the superior vena cava and right atrium. The purpose of the “port” is to deliver intravenous medications and products directly into the bloodstream and to allow for frequent blood draws [1]. Central venous access ports can be removed surgically in the operating room or endovascularly in the interventional radiology suite. Port removal involves making an incision overlying the port reservoir, dissection to the reservoir and attached catheter, removal of any anchoring sutures, gentle traction to remove the catheter, and closure of the venotomy and underlying tissue [2]. Rare, but possible complications include spontaneous catheter fracture and retained catheter [3]. If one of these complications occurs, the endovascular route is typically pursued. This entails using a guidewire to endovascularly retrieve the catheter [4].

In this paper, we present the case of a retained central venous access catheter in an adult. Interestingly, the indwelling time was only six months. Upon further review of imaging, the catheter appeared to have spontaneously fractured prior to attempted removal in the operating room. We propose that reviewing any existing chest x-rays (CXRs) or obtaining a preoperative CXR prior to port removal become the standard of care in specific patient populations. This includes patients with long indwelling catheter time, greater than two years, and those who present with concern for port infection. Preoperative imaging may ultimately change the method of retrieval from surgical to endovascular at the index operation, potentially saving the patient and hospital from the cost of additional operating room fees, anesthesia, and increased length of hospital stay.

## Case presentation

**Figure 1:**
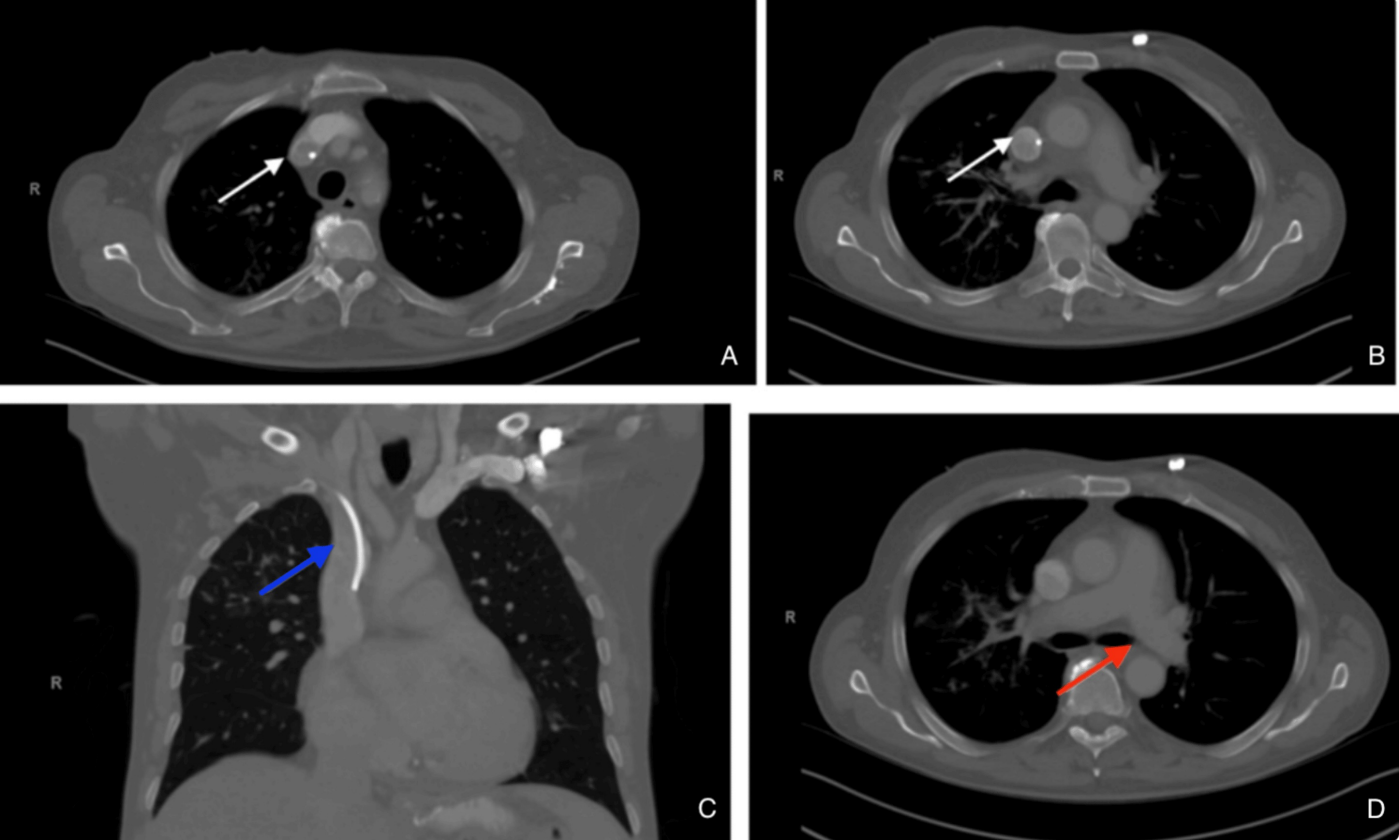
CT chest demonstrating appropriate placement of the catheter A and B: catheter within the right subclavian vein (marked by a white arrow); C: intact catheter terminating in the correct location near the cavoatrial junction (marked by a blue arrow); D: no evidence of a fractured piece of catheter within the left pulmonary artery (marked by a red arrow).

The patient is a 69-year-old male with a history of gastric adenocarcinoma status-post total gastrectomy with Hunt-Lawrence jejunal pouch who underwent placement of a right subclavian central venous access port (9.6F, Bard Medical, NJ, USA) for adjuvant chemotherapy and total parenteral nutrition due to cancer recurrence. Six months later, he presented to the emergency department with erythema, pain, and itching around the port site. Interestingly, an outpatient CT chest three days prior to presentation showed the central venous access catheter terminating within the superior vena cava without any evidence of fracture (Figure 1).

**Figure 2:**
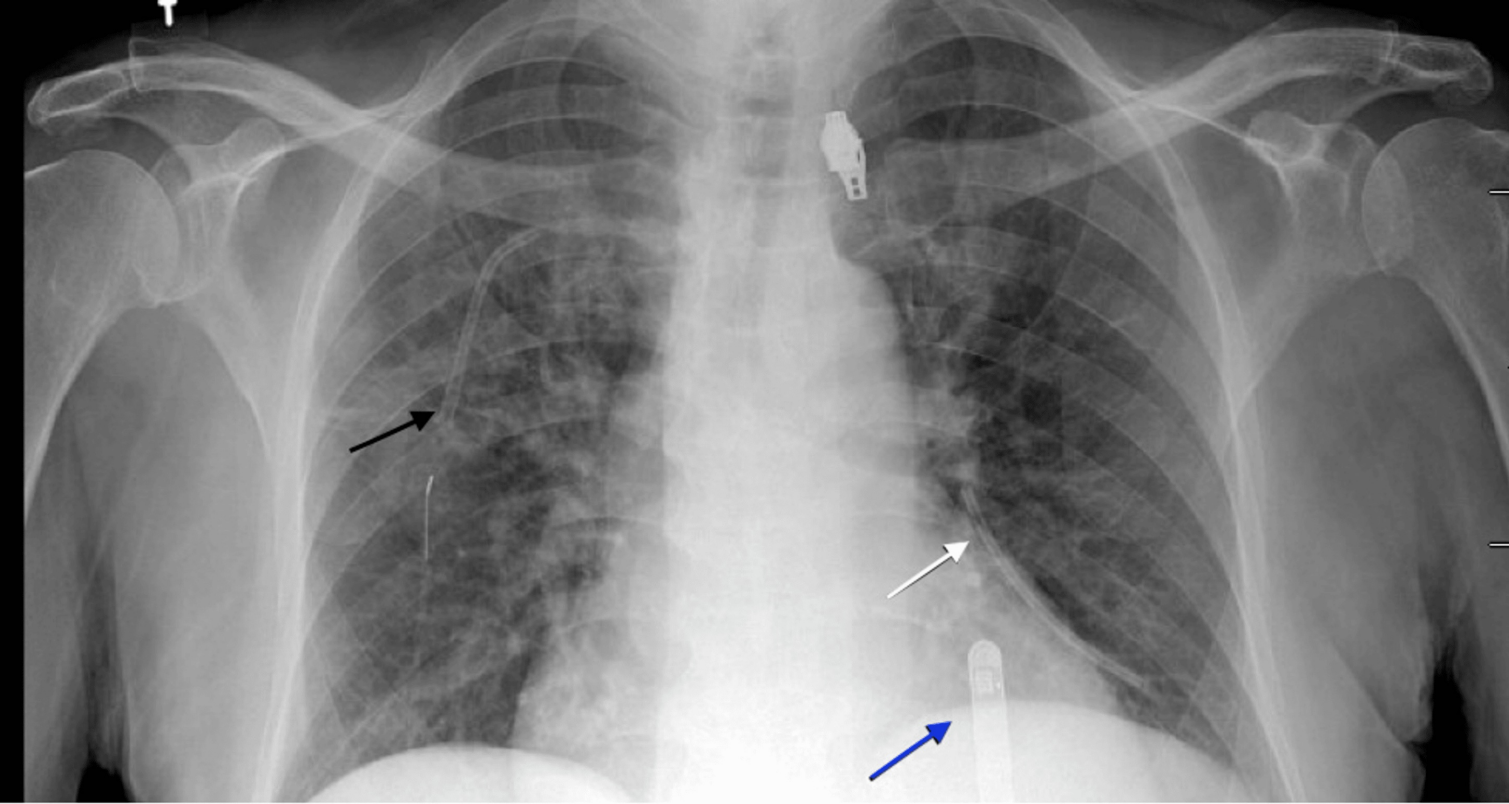
Preoperative chest X-ray Demonstrating the proximal central venous access catheter within the right subclavian vein (marked by a black arrow) and the distal fragmented catheter piece within the left pulmonary artery (marked by a white arrow). Existing loop recorder is in the appropriate position (marked by a blue arrow).

**Figure 3:**
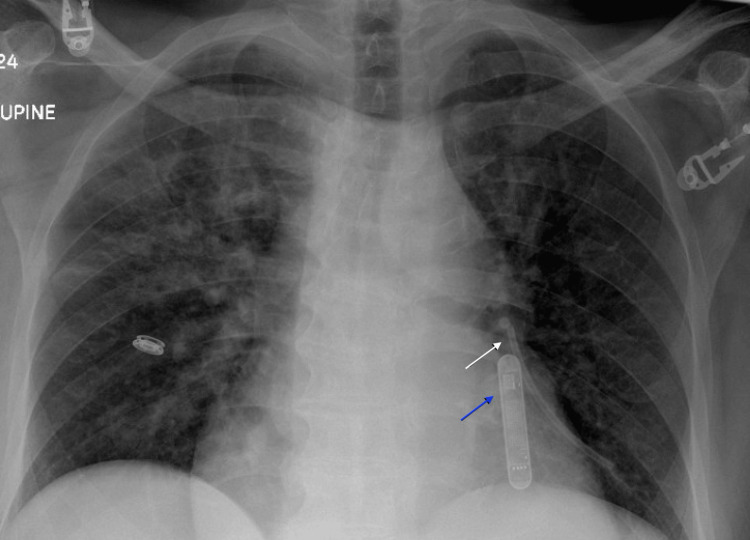
Postoperative chest X-ray Showing the interval removal of the catheter within the right subclavian vein. Presence of a retained catheter in the left pulmonary artery (marked by a white arrow). Preexisting loop recorder in the correct location (marked by a blue arrow).

**Figure 4:**
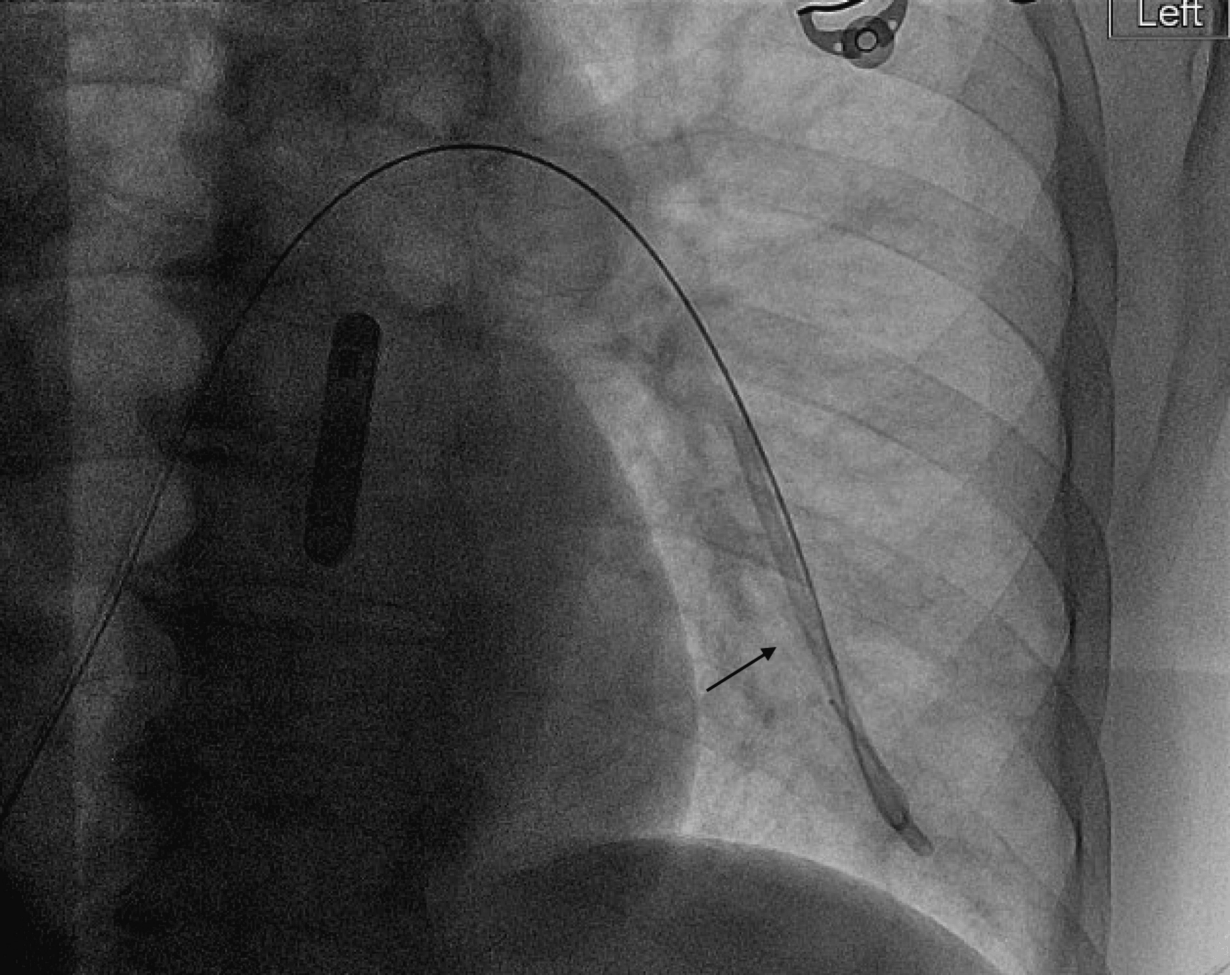
Interventional radiology percutaneous retrieval of a retained catheter in the left pulmonary artery (marked by a black arrow) via right femoral vein access

**Figure 5:**
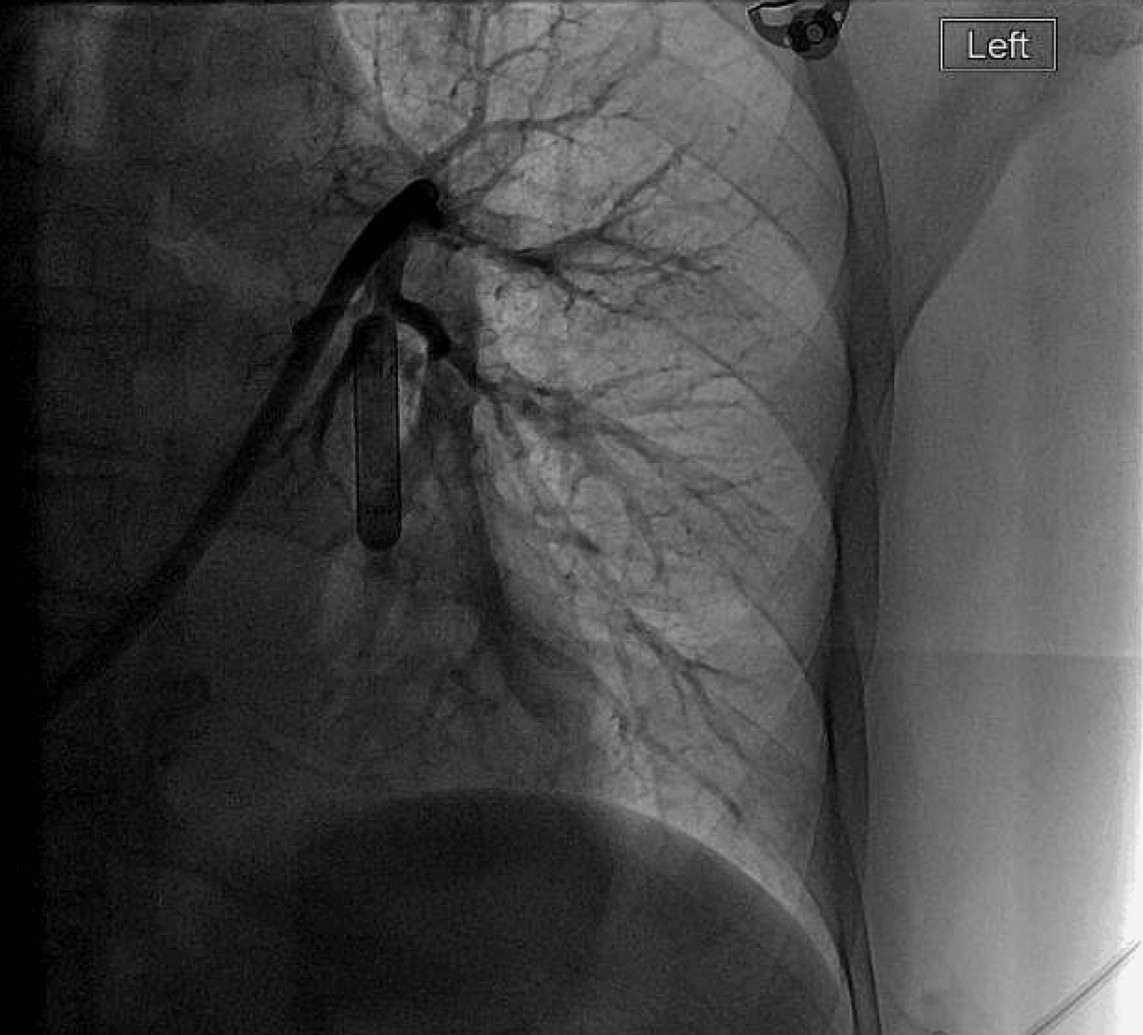
Final pulmonary arteriogram showing complete catheter fragment removal No injuries to the left pulmonary artery branches.

In the emergency department, a CXR was read as “unremarkable” (Figure 2). The following day, he was taken to the operating room for removal of his central venous access port due to concern for port site infection. Intraoperatively, there appeared to be no apparent complications. The mediport and catheter were removed easily without any aggressive traction. However, upon further inspection of the catheter tip, there appeared to be approximately 9 cm of catheter missing. A postoperative CXR was obtained and read as “retained piece of catheter in the left pulmonary artery” (Figure 3). The patient was taken the same day for successful percutaneous retrieval by interventional radiology via access through the right common femoral vein (Figure 4). A final pulmonary arteriogram demonstrated complete catheter fragment removal (Figure 5).

On retrospective review of imaging, the CXR obtained in the emergency department the day prior demonstrated that the catheter was dislodged within the pulmonary artery prior to taking the patient to the operating room. In our case, a review of preexisting imaging was not performed. Had we reviewed the preoperative CXR or obtained one prior to surgery, we would have sent the patient to interventional radiology instead of the operating room. Thus, avoiding additional use of resources, unnecessary anesthesia, and healthcare funds.

## Discussion

Complications of central venous access ports include pneumothorax, spontaneous fracture, migration of the catheter, and even thrombosis of the vein [3]. Spontaneous fracture is exceedingly rare, reported at less than 2% [5,6]. Fracture may be attributed to the material of the catheter used (polyurethane having a higher risk of fracture), location, type of chemotherapy, and indwelling time [7]. The highest amount of compression and friction is at the space between the clavicle and first rib, also known as the costoclavicular space. This area is where spontaneous fractures most often occur. On CXR, a “pinch-off sign” (POS) may be seen where the catheter is indented as it passes beneath the clavicle. This was first characterized on CXR in 1984 by Aitken and Minton [8]. In a collective review of the literature, it was found that on average, POS occurs 5.3 months after insertion but ranges from immediately to 60 months after insertion. If the subclavian vein is used as the access point, it is recommended to cannulate lateral to the midclavicular line and obtain an interval CXR periodically to rule out POS. Specifically, one, three, and five months after placement [9]. In 1990, Hinke et al. expanded on this by defining "pinch-off syndrome," which is further classified into grades 0-3. Grade 0 is described as a smooth catheter course with no evidence of narrowing, grade 1 as a catheter course with any degree of bending or deviation but no luminal narrowing, grade 2 as some degree of luminal narrowing as it passes under the clavicle, defined as a "POS," and grade 3 as showing catheter transection between the clavicle and first rib with embolization [10]. On CXR, look for catheter migration, deviation, or the “POS,” which may allude to an impending fracture.

Retained catheters in the adult population are also rare, estimated at less than 1% [11]. In the majority of cases, a prolonged indwelling time is the main contributing factor. In a paper published by the American College of Surgeons, a duration greater than two years was considered to be the most critical risk factor for retained catheter [12]. Over time, the catheter may become fixated to the wall of the vein, forming a calcified fibrin sheath. On CXR, a fibrin sheath “cast” may be seen [13]. It is imperative to avoid forceful manual traction and unnecessary dissection, as this exponentially increases the risk of fracturing the catheter and dislodging the distal end. 

Clinical symptoms of fracture or migration of the catheter include pain or swelling of the arm, chest discomfort, paresthesia, cardiac arrhythmias if it migrates into the atrium, and the inability to withdraw blood or flush the port easily [14]. Many times, patients are asymptomatic and will have a silent embolization that is found incidentally [15]. If the catheter fractures, it is imperative to remove it before a terrible complication, such as myocardial perforation, vein thrombosis, or pseudoaneurysm formation, occurs. In our case, the patient presented with pain and erythema around his port site. Final pathology and culture showed no growth, suggesting that his symptoms were secondary to a spontaneous fracture rather than infection. A CT chest three days prior confirmed the proper location of the catheter tip within the cavoatrial junction (Figure 1). The port appears to have spontaneously fractured sometime within those three days prior to hospital presentation, as the CXR in the emergency department showed catheter fragments within both the subclavian vein and the left pulmonary artery.

Early removal of central venous access ports is recommended. Removal is typically via surgical intervention. However, certain cases warrant percutaneous endovascular removal. Our case highlights the importance of having a high index of suspicion for spontaneous catheter rupture and for reviewing existing preoperative imaging prior to surgical intervention. If no imaging exists and suspicion remains high, we suggest obtaining a preoperative CXR to ensure the patient has the correct method of retrieval on the index operation.

## Conclusions

Spontaneous central venous access catheter fracture and retained catheter remain relatively uncommon. Timely removal of central venous access ports when they are no longer in use and selecting the appropriate method of removal for ports with long indwelling times are important factors to consider. Consideration of preoperative CXR should be made in patients where a high index of suspicion for spontaneous fracture exists and for those who have an increased risk of port retention. Preoperative imaging may ultimately change the extraction technique at the index operation, saving the patient from unnecessary procedures and potentially saving the hospital unnecessary costs associated with the need for these additional procedures.
